# Measuring the burden of nosocomial infection in cancer patients: an analysis based on propensity score matching in China

**DOI:** 10.3389/fpubh.2025.1647455

**Published:** 2025-11-26

**Authors:** Qingqing Tian, Yi Ding, Jiayang Tang, Anran Liu, Hui Wang, Weiwei Yang

**Affiliations:** 1 Infection Management Office, Sichuan Clinical Research Center for Cancer, Sichuan Cancer Hospital & Institute, Sichuan Cancer Center, University of Electronic Science and Technology of China, Chengdu, Sichuan, China; 2 Department of Respiratory Medicine, The Second Hospital of Jilin University, Changchun, Jilin, China

**Keywords:** hospital-acquired infections, propensity score matching, length of hospital stay, hospitalization cost, infection control

## Abstract

**Introduction:**

Cancer patients are more susceptible to nosocomial infections due to the suppression of their immune system as a result of factors such as the disease itself and treatment modalities. Nosocomial infections have become an important factor affecting the therapeutic effect, prognosis and quality of life of cancer patients, increasing their suffering and economic burden. This study aims to investigate the impact of hospital-acquired infections on the length of stay and hospitalization costs for cancer patients, providing economic health support for the prevention and control of such infections.

**Materials and methods:**

We extracted data on the basic information, infection status, hospitalization costs, and length of stay of inpatients from a large specialized cancer hospital’s infection information system and inpatient information, from July 2021 to June 2022. The influencing factors on hospitalization costs and length of stay for cancer patients were determined through literature review. After matching using the propensity score method, we analyzed the impact of hospital-acquired infections on the length of stay and hospitalization costs.

**Results:**

During the study period, a total of 407 hospital-acquired infections were reported, with an incidence rate of 0.58%. After propensity score matching and balance testing, compared with the control group, hospital-acquired infections prolonged the length of stay by 7 days (*p* < 0.01) and increased hospitalization costs by $3578.95 (*p* < 0.01).

**Conclusions and relevance:**

Hospital-acquired infections significantly increase the length of stay and hospitalization costs for cancer patients, adding to the economic burden of the disease. The use of a literature review to determine covariates makes this conclusion more scientific.

## 1 Introduction

Hospital-acquired infections (HAIs), also known as nosocomial infections, refer to infections acquired by patients or healthcare workers during hospitalization or the course of work in a hospital setting (1–3). HAI burden studies are widespread globally (4, 5). Existing research indicates that HAIs lead to an increase in patients’ length of stay and costs, imposing a serious economic burden on society (6, 7). In 2009, the U. S CDC reported that the direct medical costs of HAIs in U. S hospitals amount to $6.65 billion annually (8). A 2011 WHO report estimated that Europe incurs up to 7 billion euros annually in direct medical costs from HAIs (9). A study estimated that the direct economic burden attributable to HAIs in China exceeds 12 billion yuan (10). Some studies show that each occurrence of a hospital-acquired infection increases hospitalization costs by $2,132.00 to $15,018.00 (11, 12). Another study found that hospital-acquired infections (HAIs) resulted in additional total costs ranging from $9,310 to $21,013 and prolonged hospital stays of 5.9 to 9.6 days (13).

Propensity Score Matching (PSM) is a commonly used method that matches treatment and control group individuals based on propensity scores, reflecting outcome differences through the calculation of the average treatment effect of the two groups (14). Rosenbaum and Rubin (15) initially proposed the propensity score (PS) to evaluate the conditional probability of a research object entering the treatment group in the presence of confounding factors. PSM transforms multiple variables into an intermediate variable, not focusing on the specific value of each confounding factor that needs to be controlled, but rather on the propensity value predicted after incorporating these variables into a logistic regression equation. As long as the propensity score matches, all necessary confounding factors will be considered. This method can overcome obstacles in traditional multivariate analysis methods such as the variable not meeting the assumption of normality, multicollinearity issues, too many confounding factors, and unclear causal relationships among variables in observational studies.

Given the small number of patients with hospital-acquired infections (16), it’s worth contemplating the scientific validity of directly comparing these patients with non-infected patients and the differential impact on the length of stay and costs (17). When utilizing retrospective data for analyzing the disease burden or health economics of hospital-acquired infections, the choice of controls and the control of confounding factors become key factors influencing outcome indicators (5). In general studies, researchers often find it challenging to directly explore the net effect between independent and dependent variables due to numerous confounding factors (18).

Numerous studies have used propensity-score matching (PSM) to quantify the burden of HAIs (19); however, the resulting estimates vary widely. Apart from institutional differences, a major source of heterogeneity is whether covariates were selected appropriately, yet most economic-burden studies employing PSM provide no account of how covariates were chosen. Using a tertiary cancer hospital as an example, we applied PSM to estimate the excess economic burden attributable to HAI and explicitly report the covariate-selection process, thereby enhancing the transparency and validity of our findings.

## 2 Materials and methods

### 2.1 Data sources

This study obtained infection and hospitalization information for 69,986 inpatients from a 2057-bedded cancer hospital in Southwest China, who were discharged between Jul 2021 and June 2022 inclusive.

Hospital-acquired infection data collected for this study come from the real-time monitoring platform of the assessed hospital’s infection control department. The infection control personnel are responsible for managing the monitoring data, which includes screening, sampling, auditing, and proofreading. The hospital has established infection monitoring procedures and standard operating procedures (SOP) to maximize the accuracy and completeness of infection case diagnoses. For the purpose of this study, we defined a hospital-acquired infection (HAI) as any infection that was not present or incubating at the time of hospital admission, but occurred 48 h or more after admission. Infection control professionals in the hospital reviewed all suspected cases, and only those meeting the standard definitions were included in the analysis. Infections associated with routine postoperative complications or expected inflammatory responses were excluded unless there was clear evidence of a new infectious process.

### 2.2 Covariates

#### 2.2.1 Method for identification of possible covariates

According to the definition of covariates related to PSM (15), combined with literature and professional knowledge theory, this study searched databases such as Wanfang, VIP, CNKI, Ovid Medline, PubMed. The search terms for databases were “tumors,” “risk factors,” “hospitalization costs,” “hospitalization days,” and their synonyms, with China being the country of publication. The search period was the past 5 years (2020–2025).

The Boolean search strategy employed the Title/Sentence (TS) field as follows: (TS = ‘neoplasm’ AND TS = ‘risk factor’ AND TS = ‘hospital cost’) OR (TS = ‘neoplasm’ AND TS = ‘risk factor’ AND TS = ‘length of stay’). Details of the databases searched are given in Table 1.

**Table 1:** Database search summary.

Databases	Number of records retrieved	Number of relevant articles	Risk factors
CNKI	118	14	Age; nutritional status; complications; tumor stage; tumor site; surgery; complications
Wanfang	489	15	Age; nutritional status; underlying diseases; tumor stage; tumor site; radiotherapy; complications; surgical
VIP	201	16	Age; surgical; disease outcome; complications; condition upon admission; method of admission; principal discharge diagnosis (malignancy type); radiotherapy; medical insurance; marital status; blood transfusion therapy; invasive procedures
Ovid Medline	3,049	5	Age; surgical; disease outcome; complications; condition upon admission; method of admission; radiotherapy; medical insurance marital status; chemotherapy; department
PubMed	3,713	5	Age; medical insurance; condition upon admission; method of admission; marital status; chemotherapy; disease outcome; department; radiotherapy

#### 2.2.2 Covariate inclusion results

After aggregating Table 1, the main determinants of hospital cost and length of stay in cancer patients were identified as: surgery, disease outcome, complications, age, condition upon admission, method of admission, principal discharge diagnosis (malignancy type), radiotherapy, medical insurance, and marital status. On the basis of data availability in the electronic medical record (EMR) system in the study hospital, we selected surgery, disease outcome, complications, age, condition upon admission, method of admission, radiotherapy, marital status, and treating department as covariates for estimating the burden of healthcare-associated infection. Variable definitions and categorizations corresponding to the EMR structure are provided in Table 2.

**Table 2:** Definition and classification of covariates.

Covariates	Definition
Surgical	Surgical: any surgical procedure performed during the current hospitalization (yes/no)
Disease outcome	Discharge outcome: cured, improved, not improved, deceased, or other
Age	Age at admission (continuous variable)
Complications	Status of complications at the time of discharge: yes or no
Method of admission	Admission source: emergency department, outpatient clinic, transfer-in, or other
Radiotherapy	Radiotherapy during hospitalization: yes or no
Marital status	Marital status: married, unmarried, divorced, or widowed
Condition upon admission	Patient’s severity status within 2 h after admission, assigned by the admitting resident and automatically coded in the EMR according to the National Health Commission (NHC) admission classification standard (dangerous, urgent, general, or other)
Department	Admitting department

### 2.3 Research methods

After covariate identification, the subsequent PSM steps were (14):

(1) Estimation of propensity scores for both the HAI and non-HAI groups;
(2) Selection of nearest-neighbor matching as the optimal algorithm;
(3) Balance diagnostics to assess covariate equilibrium between matched samples;
(4) Computation of the average treatment effect (ATE) according to the distribution of the matched data—using a two-sample *t*-test when normality was satisfied and the Wilcoxon rank-sum test otherwise.

Balance after matching was evaluated with multiple quantitative indicators and graphical methods (20):

(1) Standardized mean difference (SMD).

Smaller values indicate greater similarity; benchmarks are 0.2 (small), 0.5 (medium) and 0.8 (large) effect size.

(2) Variance ratio (VR).

VR between 0.5 and 2 implies similar variances and satisfactory balance; values outside this range signal imbalance.

(3) eCDF mean.

Mean difference in empirical cumulative distribution functions; close to 0 → comparable distributions, close to 1 → poor match.

(4) eCDF maximum.

Largest difference between the two eCDFs; close to 0 → good balance, close to 1 → poor balance.

(5) Propensity-score density plot.

Superimposed densities of treated and control units; overlapping distributions indicate balance.

(6) Propensity-score histograms.

Side-by-side histograms before and after matching; similar bin heights across groups post-matching denote successful balance.

(7) Absolute standardized mean difference (ASMD) plot.

Covariates are ranked by ASMD pre- and post-matching; vertical lines at 0.05 and 0.1 aid evaluation. The vast majority of points falling within the 0.1 threshold after matching indicates adequate covariate balance.

### 2.4 Statistical analysis

The data processing and statistical analysis for Propensity Score Matching were conducted using R language (R Version 4.2.2). Patients’ basic information and factors affecting the length of hospital stay and hospitalization costs were statistically described. The R language “Mchlt” package was used, and the “nearest” method was adopted for 1:1 matching of cases with and without hospital-acquired infections. Each covariate was tested for balance, and appropriate methods were used to analyze the effects of nosocomial infections on hospitalization days and costs based on data distribution characteristics after matching.

## 3 Results

### 3.1 General situation description

A total of 69,986 inpatients were included, with 407 cases of hospital-acquired infections (0.58%). The 69,986 inpatients involved in the study had an median age of 55 (48, 64) years, an median hospital stay of 6 (3, 9) days, and an median hospitalization cost of $1550.55 (822.65, 3494.53). The general situation is shown in Table 3.

**Table 3:** General description of disease burden measurement for hospital-acquired infections.

Category	Population distribution [number of cases (%)]
Marital status	
Unmarried	2,847(4.67)
Married	61,686(88.14)
Widowed	2,513(3.59)
Divorce	2,334(3.33)
Condition upon admission	
Dangerous	67,675(96.7)
Urgent	1819(2.6)
General	400(0.57)
Other	91(0.13)
Method of admission	
Emergency	699(0.99)
Outpatient clinic	69,194(98.87)
Transfer in	9(0.01)
Other	52(0.07)
Surgical	
No surgery	52,087(74.42)
Had surgery	17,899(25.57)
Radiotherapy	
No radiotherapy	63,051(90.09)
With radiotherapy	6,935(9.91)
Complications	
No complications	64,551(92.23)
Had complications	5,435(7.77)
Disease outcome	
Cured	6,487(9.27)
Improved	60,004(85.74)
Not improved	355(0.51)
Deceased	79(0.51)
Other	3,061(0.37)

The admitting department was included as a covariate in Table 2; however, because the patients were distributed across 41 distinct departments, this variable was omitted from this table to avoid excessive length.

## 4 Balance test results

Balance diagnostics—including standardized mean difference, variance ratio, eCDF mean, and eCDF maximum—indicated that, prior to matching, department, age, surgery, and complications were all unbalanced between the hospital-acquired infection (treated) group and the control group. After propensity-score matching, all covariates achieved satisfactory balance (Tables 4, 5).

**Table 4:** Statistical table of covariate balance test before propensity matching.

Covariate	Mean of covariates by the treated group	Mean of the covariates in the control group	Standardized mean difference	Variance ratio	eCDF mean	eCDF maximum
Department	0.08	0.01	0.56^*^	54.0041^*^	0.41^*^	0.63^*^
Age	60.55	54.81	0.41^*^	1.1720	0.06	0.22^*^
Marital status	0.00	0.01	−0.05	–	0.00	0.00
Method of admission	0.00	0.00	−0.02	–	0.00	0.00
Condition upon admission	0.00	0.00	−0.00(0.00)	–	0.00	0.00
Disease outcome	2.07	2.05	0.03	1.6367	0.04	0.08
Surgery	0.61	0.25	0.73^*^	–	0.36^*^	0.36^*^
Radiotherapy	0.13	0.10	0.09	–	0.03	0.0313
Complications	0.56(0.56)	0.07(0.55)	0.98^*^(0.01)	–	0.49^*^	0.49^*^

* represents covariates with large differences or unbalanced distributions in the two samples before matching.

**Table 5:** Statistical table of covariate balance test after propensity matching.

Covariate	Mean of covariates by treatment group	Mean of the covariates in the control group	Standardized mean difference	Variance ratio	eCDF mean	eCDF maximum
Department	0.08	0.08	0.03	1.27	0.00	0.01
Age	60.55	61.57	−0.07	1.29	0.01	0.05
Marital status	0.00	0.00	0.07	–	0.00	0.00
Method of admission	0.00	0.00	0.00	–	0.00	0.00
Condition upon admission	0.00	0.00	0.00	–	0.00	0.00
Disease outcome	2.07	2.05	0.02	1.05	0.00	0.01
Surgery	0.61	0.63	−0.04	–	0.02	0.02
Radiotherapy	0.13	0.16	−0.09	–	0.03	0.03
Complications	0.56	0.55	0.01	–	0.01	0.01

**Figure 1:**
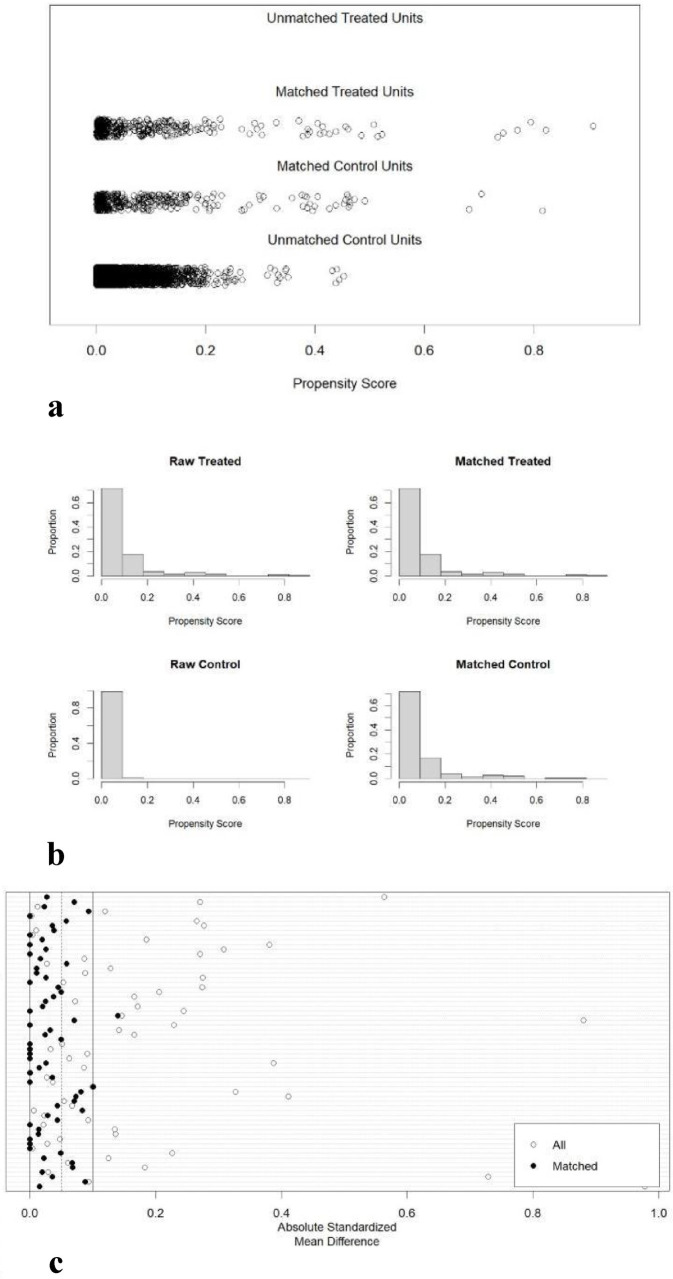
Distribution of propensity scores in the treatment and control groups. **(a)** Propensity score distribution plot; **(b)** propensity score histogram; **(c)** absolute standardized mean difference dot plot.

The distribution of propensity scores (Figure 1a) showed that, pre-matching, the treated and control group lacked similar distributional shape and location, indicating imbalance. Post-matching, the two distributions became almost superimposable, demonstrating that balance was achieved. The histograms of propensity scores (Figure 1b) revealed inconsistent patterns between groups before matching; after matching, the histograms were nearly identical, confirming good balance in covariate distributions across propensity scores. The absolute standardized mean difference plot (Figure 1c) displays the post-matching standardized mean differences for each covariate; virtually all “matched” points fall within 0.1, indicating excellent covariate balance after matching.

## 5 Results of disease burden measurement for hospital-acquired infections

**Figure 2:**
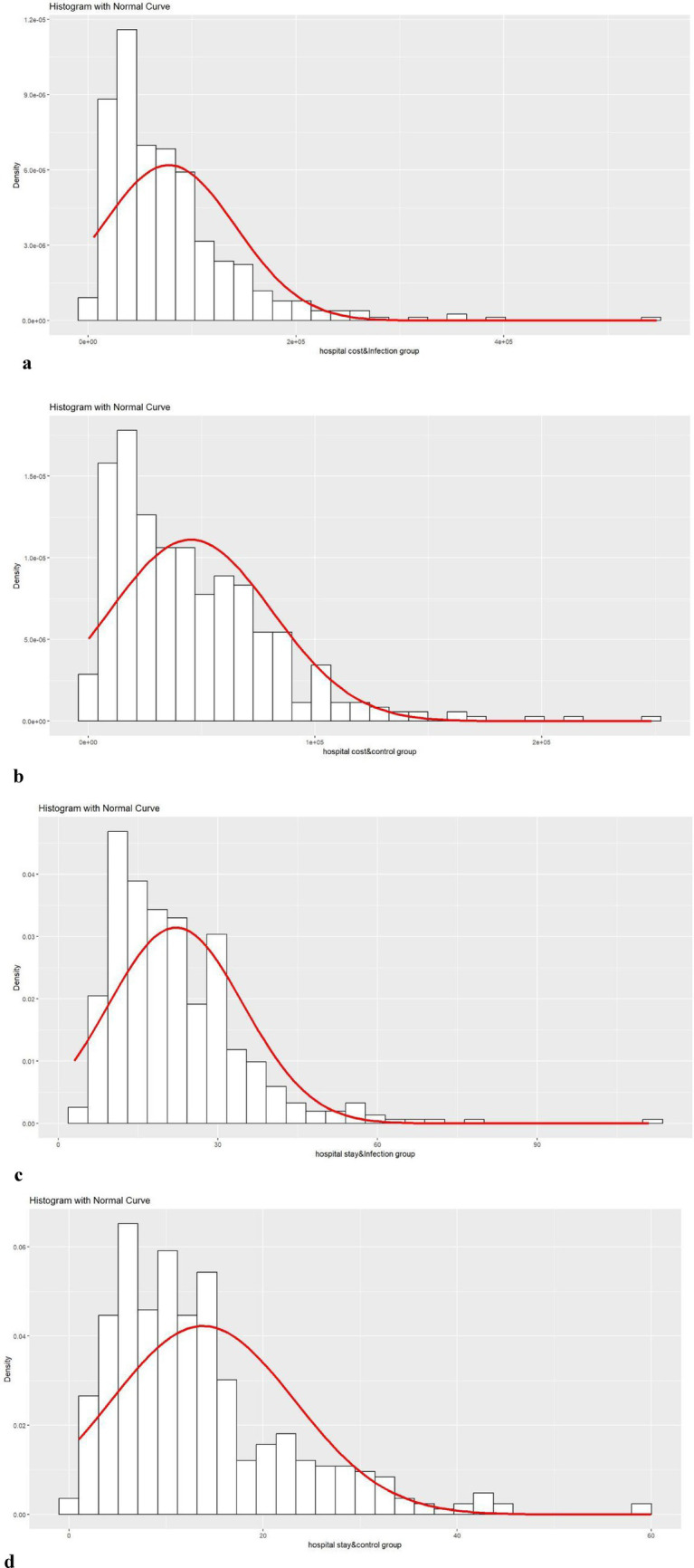
Post-matching distribution plots by group. **(a)** Post-matching distribution plot for hospital cost of infection group. **(b)** Post-matching distribution plot for hospital cost of control group. **(c)** Post-matching distribution plot for hospital stay of infection group. **(d)** Post-matching distribution plot for hospital stay of control group.

The length of hospital stay and hospitalization costs after matching exhibited a skewed distribution, as shown in Figure 2. Therefore, the Wilcoxon rank-sum test was used to analyze the differences between the two groups. Compared to the control group, the hospital-acquired infection group had a prolongation of hospital stay by 7 days (*p* < 0.01) and an increase in hospitalization costs by $3578.95 (*p* < 0.01), as shown in Table 6. The occurrence of hospital-acquired infections significantly increases the length of hospital stay and hospitalization costs.

**Table 6:** *W* rank sum test result table of hospital costs and hospital days (after matching).

Item	Group	Median	Interquartile range	*W* value	*p*-value
Hospital cost	Nosocomial infection group	$8736.17	(4649.85, 27661.63)	112,079.00	<0.01
	Control group	$5157.22	(2518.42, 22939.58)		
Hospital day	Nosocomial infection group	19	(13, 28)	−120,897.00	<0.01
	Control group	12	(7, 17)		

## 6 Discussion

The burden of hospital-acquired infections (HAIs) has become a pressing issue in modern healthcare systems. Our study provides a comprehensive analysis of the economic impact of HAIs on cancer patients in a specialized oncology hospital in China. We found that HAIs significantly increased hospital length of stay and hospitalization costs. This finding is consistent with previous studies conducted in different countries and healthcare settings. Several studies have reported similar results. For example, Huang L et al. showed that cancer patients with hospital-acquired infections (HAIs) had higher hospitalization costs (i.e., $16,927) and longer length of stay (LOS) (i.e., 22 days) compared with the non-HAI group. HAIs resulted in additional hospitalization costs and prolonged length of stay of $4,919 and 9 days, respectively (21).

The propensity score matching (PSM) method was used in our study to more accurately estimate the economic burden of HAIs by controlling for a variety of confounders that may affect hospitalization costs and length of stay. The propensity matching method has been widely used in health econometric studies to address selection bias and confounders. For example, PSM was used to assess the impact of HAIs on health care delivery and survival of cancer patients in a study by Liu C et al. They found that HAIs significantly lengthened hospital stays, increased hospital costs, and worsened cancer patient survival, highlighting the importance of controlling for confounding factors in such analyses (22). Our study also emphasizes the need for careful selection of covariates when using PSM. Through a comprehensive review of the literature, we identified key factors that may influence hospitalization costs and length of stay for cancer patients. This approach ensured that the covariates included in the PSM model were relevant and comprehensive. In contrast, some previous studies may have underestimated the financial burden of HAIs by omitting important covariates. As noted by Orlando S et al., the inclusion of appropriate covariates is essential to obtain reliable estimates of the economic impact of HAIs (23).

In addition, our study highlights the significant economic burden of HAIs on cancer patients during the COVID-19 pandemic. Pandemics place additional strain on the health care system, and the increased incidence of HAIs may exacerbate this burden (24). Studies conducted during pandemics have made similar findings. For example, a study by Sentiff et al. (25) found that HAIs in patients with COVID-19 led to prolonged hospitalization and increased healthcare costs.

However, there are some limitations to our study, which should be recognized. First, the study was conducted in only one oncology hospital, which limits the generalization of the findings to other healthcare institutions. Second, due to the complexity of factors associated with the diagnosis of cancer patients, we were unable to include all potential covariates in the PSM analysis. Future studies could consider using more advanced statistical methods or machine learning algorithms to handle more covariates and improve the accuracy of the estimation. Third, the data used in this study were collected retrospectively, which may introduce some bias despite the use of PSM. A prospective study using a well-designed data collection program could provide more reliable evidence on the economic burden of HAIs.

In summary, our study provides valuable insights into the economic impact of HAIs on Chinese cancer patients. These findings emphasize the importance of implementing effective infection prevention and control measures to reduce the burden of HAIs and improve patient prognosis. Future studies should continue to explore innovative approaches and expand the scope of analyses to better understand the economic burden of HAIs in different healthcare settings.

## 7 Conclusion

Focusing on the period from July 2021 to June 2022 of the COVID-19 pandemic, this study used propensity score matching to accurately assess the burden of disease due to hospital-acquired infections in a specialist oncology hospital. The literature review method was innovatively used to rigorously select covariates to ensure the scientific validity and accuracy of the measurement. The results showed that the excess hospitalization costs measured in this study were higher than some of the established studies, suggesting that some of the previous studies may have underestimated the economic burden of hospital-acquired infections.

Our results may help more accurately estimate the economic burden of disease caused by hospital-acquired infections in cancer patients, providing data support for subsequent health economic research on hospital-acquired infections, and a reference for improving covariate inclusion in such research.

## Data Availability

The datasets generated and/or analyzed during the study are available from the corresponding author upon reasonable request.
