# Supplementary figures and images for: Measuring the burden of nosocomial infection in cancer patients: an analysis based on propensity score matching in China
Source: Front Public Health. 2025 Nov 26;13:1647455. doi: 10.3389/fpubh.2025.1647455 (PMC12689532; doi:10.3389/fpubh.2025.1647455)

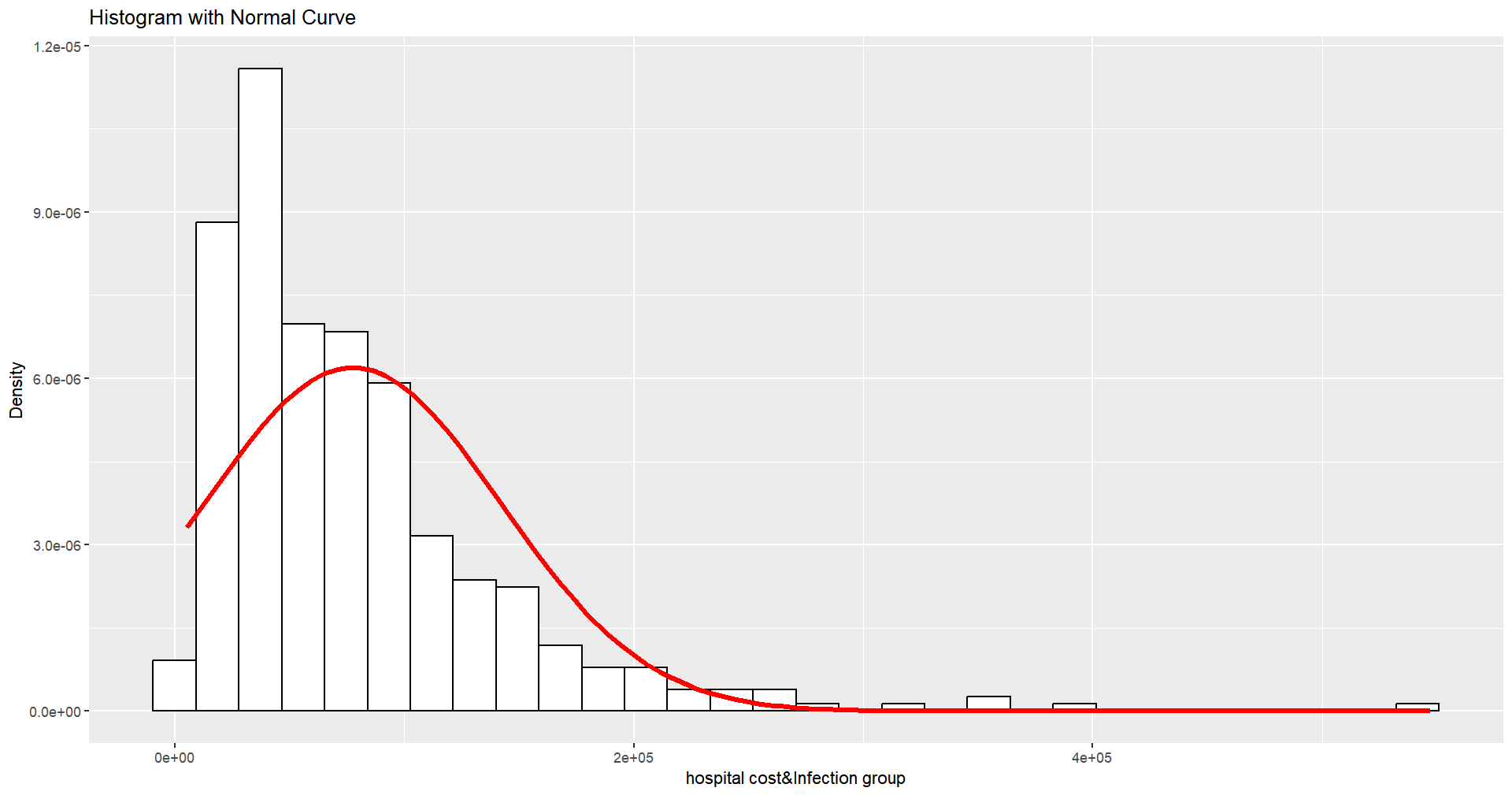

Supplement: Supplementary file 1 [file Data_Sheet_1.ZIP › 补充材料/hospital cost&Infection group.png]

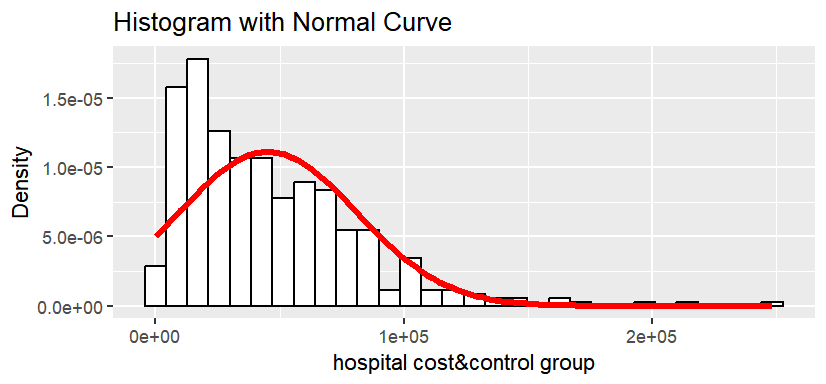

Supplement: Supplementary file 1 [file Data_Sheet_1.ZIP › 补充材料/hospital cost&control group.png]

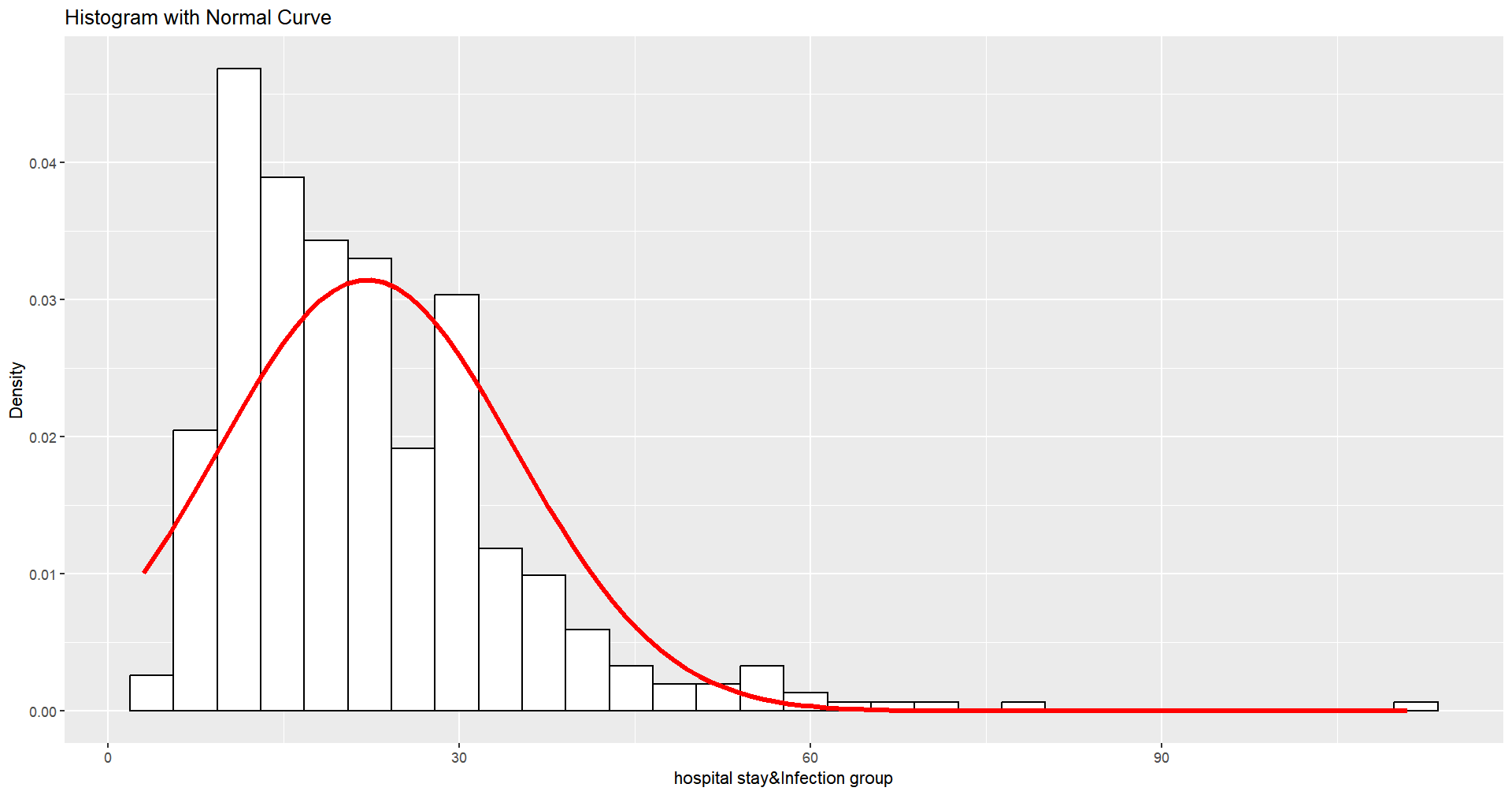

Supplement: Supplementary file 1 [file Data_Sheet_1.ZIP › 补充材料/hospital stay&Infection group.png]

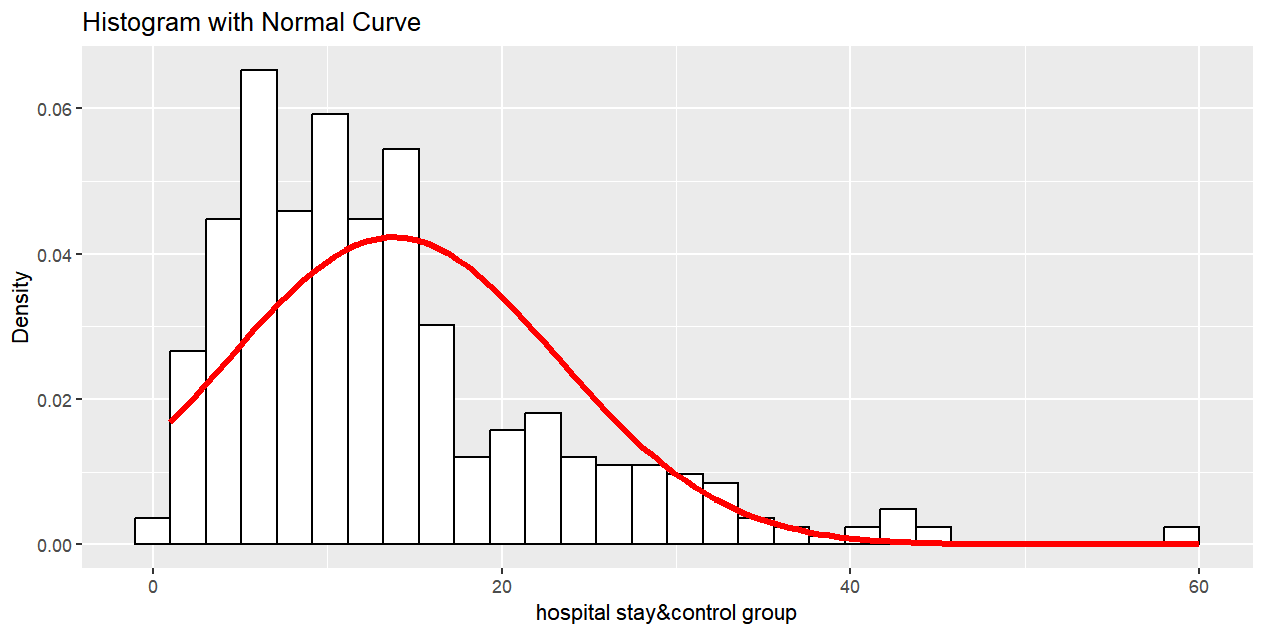

Supplement: Supplementary file 1 [file Data_Sheet_1.ZIP › 补充材料/hospital stay&control group.png]
